# Partial Least Square Discriminant Analysis Based on Normalized Two-Stage Vegetation Indices for Mapping Damage from Rice Diseases Using PlanetScope Datasets

**DOI:** 10.3390/s18061901

**Published:** 2018-06-11

**Authors:** Yue Shi, Wenjiang Huang, Huichun Ye, Chao Ruan, Naichen Xing, Yun Geng, Yingying Dong, Dailiang Peng

**Affiliations:** 1 Key Laboratory of Digital Earth Science, Institute of Remote Sensing and Digital Earth, Chinese Academy of Science, Beijing 100094, China; shiyue@radi.ac.cn (Y.S.); yehc@radi.ac.cn (H.Y.); m17611337552@163.com (C.R.); xingnc@radi.ac.cn (N.X.); gengyun@radi.ac.cn (Y.G.); dongyy@radi.ac.cn (Y.D.); pengdl@radi.ac.cn (D.P.); 2 University of Chinese Academy of Sciences, Beijing 100049, China; 3 Key Laboratory of Earth Observation, Sanya 572029, China; 4 State Key Laboratory of Remote Sensing Science, Institute of Remote Sensing and Digital Earth, Chinese Academy of Sciences, Beijing 100094, China; 5 School of Electronics and Information Engineering, Anhui University, Hefei 230601, China

**Keywords:** high spatial resolution, PlanetScope, feature extraction, damage mapping, rice dwarf, rice blast, glume blight

## Abstract

In recent decades, rice disease co-epidemics have caused tremendous damage to crop production in both China and Southeast Asia. A variety of remote sensing based approaches have been developed and applied to map diseases distribution using coarse- to moderate-resolution imagery. However, the detection and discrimination of various disease species infecting rice were seldom assessed using high spatial resolution data. The aims of this study were (1) to develop a set of normalized two-stage vegetation indices (VIs) for characterizing the progressive development of different diseases with rice; (2) to explore the performance of combined normalized two-stage VIs in partial least square discriminant analysis (PLS-DA); and (3) to map and evaluate the damage caused by rice diseases at fine spatial scales, for the first time using bi-temporal, high spatial resolution imagery from PlanetScope datasets at a 3 m spatial resolution. Our findings suggest that the primary biophysical parameters caused by different disease (e.g., changes in leaf area, pigment contents, or canopy morphology) can be captured using combined normalized two-stage VIs. PLS-DA was able to classify rice diseases at a sub-field scale, with an overall accuracy of 75.62% and a Kappa value of 0.47. The approach was successfully applied during a typical co-epidemic outbreak of rice dwarf (Rice dwarf virus, RDV), rice blast (*Magnaporthe oryzae*), and glume blight (*Phyllosticta glumarum*) in Guangxi Province, China. Furthermore, our approach highlighted the feasibility of the method in capturing heterogeneous disease patterns at fine spatial scales over the large spatial extents.

## 1. Introduction

Global change has already caused severe co-epidemics in rice, including rice dwarf, rice blast, glume blight, and sheath blight [1,2]. These threats may result in serious deterioration of grain yield and quality [3,4,5]. Traditionally, manual scouting has been the only way to detect and discriminate crop pests and diseases, but these investigations are expensive and time-consuming [6]. Real-time characterization, identification, and classification of different diseases are necessary to mitigate the problems associated with diseases infestations monitoring and pesticide overuse. Satellite-derived multi-spectral imagery is a feasible method for disease detection and assessment [7]. Satellite-based imagery is routinely captured and represents an independent, affordable source of data for large-scale monitoring of crop diseases [8,9,10]. This development has made precision field observations possible for large areas, which helps agricultural administrations determine whether to implement price regulation or to provide financial subsidies to damaged regions. Additionally, knowing the spatial extent of damage caused by crop diseases is vital to agricultural insurance companies. 

In practice, the use of satellite-derived imagery to monitoring co-epidemic diseases at sub-field scales must address two constraints. Firstly, different diseases can cause similar stresses and symptoms (e.g., discoloration, wilting, and rot), which can result in confusion for multiple disease detection using a single-date satellite imagery. Secondly, different objects with similar spectral properties are affected by a mixed pixel effect from moderate resolution sensors [11]. Thus, canopy structural characteristics and the biological effects induced by different diseases often vary at fine spatial scales (<5 m). Furthermore, disease discrimination and mapping using conventional coarse- to moderate-resolution (≥15 m) satellites (e.g., Landsat OLI-8, Sentinel-2), are too coarse to capture the effects caused by crop diseases on an agroecological system [12]. One approach to overcome that has recently been developed to overcome such limitations is the use of multi-temporal hyperspectral or multi-spectral satellite data. Hyperspectral data with a moderate spatial resolution are capable of partly overcoming the limitation of mixed pixels, but the high-cost and data redundancy in time-series analysis limits their use in crop stress monitoring. Alternatively, constellations of very high spatial resolution (VHSR) nano-satellites are composed of a series of small, compact sensor units (~10 kg) that are typically cheap and replaceable. These nano-satellites can fundamentally change spatial analysis methods for crop damage mapping [13]. They can also enhance the opportunity for agricultural monitoring and damage assessment. For instance, the pixels at metric resolutions are directly related to the pathological appearance of crop diseases. Additionally, the information gathered on within-field variability in growing conditions and disease infestations is important for precision agriculture. Planet Labs Inc. (www.planet.com) based in San Francisco, United State, operates the largest nano-satellite constellation system, with a satellite flock termed “PlanetScope” that collects multi-spectral imagery at a 3-m spatial resolution with four bands, including blue, green, red, and NIR. Houborg et al. [14] employed a data mining approach to build a set of rule-based regression models that relate Planet imagery to atmospherically corrected Landsat-8 Normalized Difference Vegetation Index (NDVI), and successfully applied the method for a desert agricultural landscape in Saudi Arabia. Kääb et al. [15] used the PlanetScope scenes before and after the earthquake for observation of land surface displacements; Baloloy et al. [16] assessed and compared the biomass predictor potential of vegetation indices derived from the Sentinel-2, RapidEye, and PlanetScope satellite systems.

The potential application of VHSR data spans across the diverse aspects of vegetation observation [17], and provides continuous spatio-temporal information for characterizing plant conditions [18,19,20]. It is known that plants will exhibit specific host-pathogen interactions while under different infestations, such as, reductions in leaf area, variation in pigment content, and destruction of canopy morphology [21]. These effects can be obtained by continual spectral responses in the visible and near-infrared bands from multi-temporal observations [22,23]. A number of broadband vegetation indices have been developed to capture plant biophysical, structural, and physiological parameters based on spectral analysis at the canopy scale [24,25,26,27]. Zhang et al. [23] successfully developed a multi-temporal, modified soil-adjusted vegetation index (MSR) on the HJ-CCD images for detecting and mapping armyworm outbreak in Tangshan, Hebei Province of China. Yuan et al. [28] assessed the performance of the most suitable multi-spectral vegetation indices, GNDVI and NDVI, using Quickbird data for monitoring yellow rust disease in winter wheat. The results produced an acceptable accuracy for mapping yellow rust damage caused by disease. Apart from the direct utilization of vegetation indices, various automatic or semi-automatic methods have exhibited potential for these statistical learning-based models in agricultural research [29,30,31,32]. Gil et al. [33] used IKONOS imagery to test the performance of Support Vector Machines, Artificial Neural Networks (non-parametric methods), Mahalanobis Distance, and Maximum Likelihood (parametric methods) in damage detection, and to map the spread of an aggressive invasive alien species in the Azorean Laurel Forest. White et al. [34] assessed the effectiveness of using SPOT-5 10-m multispectral imagery and a logistic regression model to detect and map red-attack damage for an area near Cranbrook, British Columbia, Canada. These studies have demonstrated that a pixel-based method can be used to detect symptoms induced by stresses using VHSR data. However, the opportunities for the use of VHSR satellite data and methods for discrimination of multiple disease species have rarely been explored. This is due to the fact that selecting spectral features and their inherent multicollinearity can overwhelm analytical methods and create difficulties when determining the best approach for detecting crop diseases. For example, Franke et al. [35] examined the potential of multi-spectral remote sensing for a multi-temporal analysis of crop diseases, and used mixture tuned matched filtering (MTMF) on the results. Additionally, they used the NDVI calculated from QuickBird images to classify different species of leaf rust and powdery mildew. While successful, their approach was only moderately suitable for disease detection due to the homogeneity of the selected spectral features. Therefore, it is essential to develop a novel classification method that can fully address the similarity in spectral features among different infestations.

Partial least squares (PLS) analysis is an efficient multivariate statistical technique that addresses the complexities associated with multicollinearity by simultaneously executing principle component extraction and classification [36,37,38]. However, PLS alone does not provide insight on the most sensitive features that may contribute to a final classification. Researchers have shown that pre-selecting features based on the variable importance in the projection (VIP) score serves as an apt measure for determining feature importance [39,40]. Furthermore, selection of important spectral features has been observed to improve model performance [41]. To our knowledge, there are very few studies that assess the utility and potential of PLS for regression and classification. Additionally, there has been little effort to explore whether the pixel-based, PLS-based discriminant algorithm obtained from the PlanetScope dataset can effectively and consistently map the occurrence of different crop diseases. 

Research progress and practical applications in remote sensing have motivated us to develop a novel approach for mapping damage in rice caused by different diseases. In this paper, the multiple-disease outbreak of rice dwarf, rice blast, and glume blight was selected for case study. The outbreak occurred in the rice planting area of Guanxi Province, China, during the autumn of 2017. The cloud-free, bi-temporal, high spatial resolution PlanetScope (PL) images acquired before and after the outbreak were used as a basis for analysis. The aims of this study were (1) to propose a series of normalized two-stage vegetation indices (VIs) that characterize the host-pathogen interaction of individual diseases; (2) to evaluate partial least squares discriminant analysis (PLS-DA) for mapping the spatial distribution of rice diseases using the proposed normalized two-stage VIs; (3) to produce the damage map of rice diseases at fine spatial scales. To our knowledge, this study is also the first attempt using the commercial PL datasets in precision agricultural management.

## 2. Materials and Methods

### 2.1. Study Area

**Figure 1:**
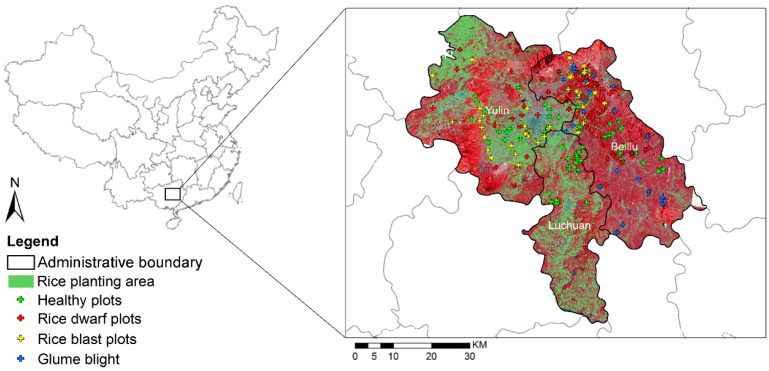
A false-color map of study areas and survey plots in Guangxi Province, China. The rice planting areas are revealed as green polygons.

A site suffering a severe infestation of rice dwarf, rice blast, and glume blight disease was selected as the study area. The outbreak occurred in the site during the autumn of 2017. The site featured a total area of over 6000 km^2^ and was located in the east of Guangxi Province, China (22°64′ N, 110°14′ E). The site was characterized by hills and mountainous terrain and featured an average elevation of 61.3 m. The study area contains three regions, including Yulin City, Beiliu City, and Luchuan County (Figure 1). Rice is a major crop in the area, planted in late-July and harvested in early-November. According to the historical records from the local Plant Protection Agency, the disease infestations are observed to always occur during late-August and late-October due to the area’s rain-fed agroecological structure.

In the study area, grasslands are the dominant natural vegetation and the land contains sections of developed surfaces (e.g., roads and buildings). The Guangxi Department of Agriculture (GXDA) annually publishes Cultivated Land Survey reports quantifying the rice crop area using shapefiles. The crop areas investigated by the GXDA are shown in Figure 1.

### 2.2. Satellite Data

The satellite data used in this case are acquired by Planet’s PlanetScope (PL) Eatrh-Imager, multispectral Cubsat constellation. Here, the PL Ortho Scenes products which are orthorectified, scaled Top of Atmosphere Radiance image (Level 3B) and are delivered as analytical (4-band) products [42]. PlanetScope captures imagery at a ground sampling distance of 3.7 m at a reference altitude of 475 km and the imagery is then orthorectified to a pixel size of 3 m [43]. The information about its specific attributes is shown in Table 1. A total of 552 cloud-free PL scenes were acquired before (21 August) and after (30 October) the occurrence of rice diseases in study area. The orthorectified scene mosaic were then printed out in the Univeral Transverse Mercator (UTM) projection to provide near complete coverage of the study area.

### 2.3. Field Investigation

**Figure 2:**
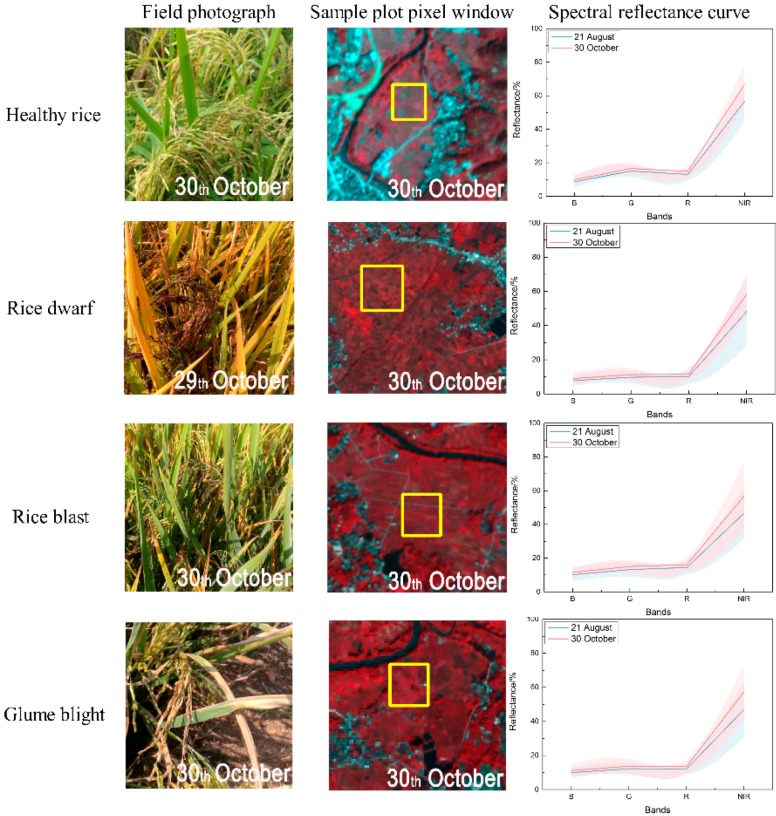
The representative samples for healthy rice and rice infested with dwarf, blast, and glume blight. Plots on the right show averaged spectral reflectance and deviation (the shadows) of each class collected on 21 August and 30 October.

In this study, a total of 250 plots were surveyed to assess the damage severity caused by crop diseases as ground truth data during 29 and 31 October 2017 (Figure 1). The sampling designs for each individual disease species were based on the Rules of Investigation and Forecast of Rice Blast (GB/T 15790-2009), Rules of Investigation and Forecast of Disease caused by Rice Black-Streaked Dwarf Virus (NY/T 2730-2015), and the Rules for Investigation and Forecast of the Rice Blight Virus (NY/T 1609-2008). Each site covered an area of 3 m × 3 m and the plots were compared with corresponding image pixels. A visual discrimination method was applied to assess the infestation status and damage severity for each plot because some rice diseases occurred simultaneously in the study area. The plots were labeled as healthy if they had not been infested or if the proportion of damaged leaves was less than 10%; for rice leaves infected with mixed infestations were defined as the corresponding disease with the most dominant proportion of the infestation. Figure 2 shows a representative example for healthy samples and other samples infected with rice dwarf, rice blast, and glume blight disease, and the corresponding PL multi-spectral reflectance curves. In this study, 70% of the plots were randomly selected for model calibration and the remaining 30% were used for validation.

### 2.4. Mapping Various Rice Disease Infested Areas

Pathologically, the progressive development between the various disease infestations are different, although these infestations may lead to similar external symptoms. There are no observable lesions in early infestation for rice dwarf, but the growth of rice becomes limited as the virus develops [44]. For rice blast, lesions may initially appear gray-green and water-soaked with the darker green borders. The lesions can expand rapidly to several centimeters in length and affect the leaf tissue and photosynthetic pigments [45]. The initial symptoms for glume blight can reveal the minute brown dots on the leaf blades and glumes. These dots later becoming cylindrical or oval as the leaf loses moisture in shape [46]. Several vegetation indices and models were employed to characterize these individual pathological features.

#### 2.4.1. Spectral Features for Mapping Diseases

Considering the potential pathological impact of disease infestations mentioned above, six vegetation indices (VIs) that related to plant growth, vegetation coverage, and radiant absorption of pigments were selected for characterizing the biophysical variations caused by individual infestation: Normalized difference vegetation index (NDVI), the soil-adjusted vegetation index (SAVI); Triangular vegetation index (TVI); Re-normalize difference vegetation index (RDVI); Modified Simple Ratio (MSR); and Structural Independent Pigment Index (SIPI). Among them, the NDVI is the best known VI for mapping the amount of green biomass in vegetation of low- to moderate density [47]; the SAVI is developed to minimize soil influences on canopy spectral, which has the potential of observing damaged fields with different proportions of soil exposure [48]; the TVI indicates the radiant energy absorption of chlorophyll [49]; the RDVI modified the NDVI to make the index more sensitive to high LAI values [50]; the MSR has great sensitivity to green LAI and resistant to atmosphere effects; and the SIPI indicates the ratio of carotenoids and chlorophyll a at the canopy scale. The definitions of these VIs in detail are listed in Table 2.

#### 2.4.2. Normalized Two-Stage Vegetation Indices

The normalized difference model was used to characteristic the progressive development of internal and external symptoms caused by rice diseases based on bi-temporal data from the PL constellation. The design of these normalized two-stage vegetation indices capitalizes on biophysical and pathological concepts and spectral features of disease infestations. The indices also isolate the properties of vegetation growth and crop biochemical change caused by disease. The vegetation indices changed in magnitude from the 21 August to the 30 October images were calculated by using the normalization quantification formula:

(1) $$VI_{two-stage}=\frac{VI_{30October}-VI_{21August}}{VI_{30October}+VI_{21August}}$$

where *VI_two-stage_* is the normalized two-stage VI change, *VI*_21 *August*_ and *VI*_30 *October*_ are the VIs extracted from the images acquired before (21 August) and after (30 October) the occurrence of disease.

#### 2.4.3. The Sensitivity of the Identified Spectral Features to Rice Diseases

We used statistical analyses in order to examine whether the proposed spectral features were sensitive to disease infestations, and to compare the performance of normalized two-stage VIs and single-date VIs for tracking individual biophysical and pathological progression. These priori knowledge-based statistical analyses were used based on two standards. Firstly, a threshold-based classification test was implemented to determine the classification capability of each spectral feature for healthy rice, and those infested with dwarf, blast, and glume blight [53]. The second criterion was to test the independence between variables. To further examine whether the satellite-derived vegetation indices are sensitive to disease infestations, a standard analysis of variance (ANOVA) was conducted to consider the impacts of information redundancy and multi-collinearity [23]. Here, ANOVA was used with a confidence level of 95% (*p*-value < 0.05) to ensure that the identified spectral features had sufficient independence and heterogeneity in subsequent analysis.

#### 2.4.4. Diseases Occurrence Mapping Using Partial Least Squares Discriminant Analysis (PLS-DA)

In the PLS-DA frame, the response variable (i.e., disease species) is binary and expresses class membership. The PLS procedure create several eigenvectors of spectral matrices which will produce scores that explain both the variance of the spectral features, as well as the correlation with the response variables [37]. It is essential to address the multi-collinearity between variables to reduce the risk of overfitting due to correlated variables in the PLS procedure [32]. Therefore, it is necessary to test and select the optimal PLS components (i.e., VIs in this study). A reliable way of testing the significance of each PLS component is cross validation. By using the candidate VIs, the parameters of the PLS-DA model are optimized based on ten-fold cross-validation, conditioned on the training dataset. 

An essential requirement for the PLS-DA model is to calculate the variable importance in the projection (VIP) score of each variable (i.e., VIs in this study). The VIP score serves as a measure of the contributions of input variables to the classification results [41]. The VIP score is defined as follows:

(2) $$VIP_{k}=\sqrt{K\sum_{a=1}^{A}\left[\left(q_{a}^{2}t_{a}^{T}t_{a}\right)\left(w_{ak}/{\left\|w_{k}\right\|}^{2}\right)/\sum_{a=1}^{A}\left(q_{a}^{2}t_{a}^{T}t_{a}\right)\right]}$$

where *VIP_k_* is the importance of the *k*th VIs, *w_ak_* is the corresponding loading weight of the *k*th VIs in the *a*th PLS-DA component, *t_a_*, *w_a_* and *q_a_* are the *a*th column vectors, and *K* is the total number of VIs (*K* = 6 in this study, as given in Table 2).

Subsequently, a new PLS-DA model was computed from the selected VIs, and then used to execute classification of rice diseases on satellite imagery. In this section, PLS-DA model development, VIP score calculations and model optimization were processed using the PLS Toolbox 8.1.1 (Eigenvector Research Incorporated) for Matlab R2017a (Mathworks, Natick, MA, USA).

## 3. Results

### 3.1. Responses of Spectral Features to Different Infestations

**Figure 3:**
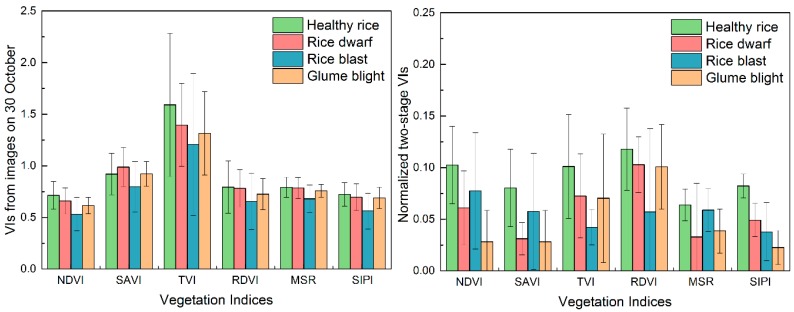
The mean and standard deviations of pixel-based single-date VIs shown on (**left**) and normalized two-stage VIs shown on (**right**).

The responses of the six VIs and normalized two-stage VIs to changes in disease are illustrated in Figure 3, where their means and standard deviations are compared at different damage levels. This comparison reveals that the normalized two-stage VIs, especially for NDVI, SAVI, MSR, and SIPI, exhibited a stronger response to the diseased samples. For healthy rice, the normalized two-stage vegetation indices revealed greater differences with the rice infested with disease compared to corresponding single-date VIs from the images on 30 October. For the diseased rice, the responses of the newly proposed normalized two-stage vegetation indices were strongly associated with the individual pathological progress of different diseases. Typically, glume blight disease resulted in leaf wilt and death of foliar tissue, which impacted canopy structure. These characteristics were captured by the normalized two-stage NDVI, MSR, and SIPI, which were sensitive to canopy morphology.

The threshold-based classification ability of each VI was tested for different diseases and pests, and the results are shown in Table 3. ANOVA results provided a quantitative measure of discriminative capability (Table 4). The results indicate that the single-date VIs could only differentiate between healthy and diseased samples but had difficulty in discriminating between different infestations, with a different significance of 0.95 for the confidence interval. In contrast, the normalized two-stage VIs exhibited more potential for observing differences among all classes (*p* < 0.05). This suggests that the normalized two-stage indices could discriminate between different rice diseases and healthy samples.

### 3.2. Mapping Disease Infestations with PL Satellite Imagery

**Figure 4:**
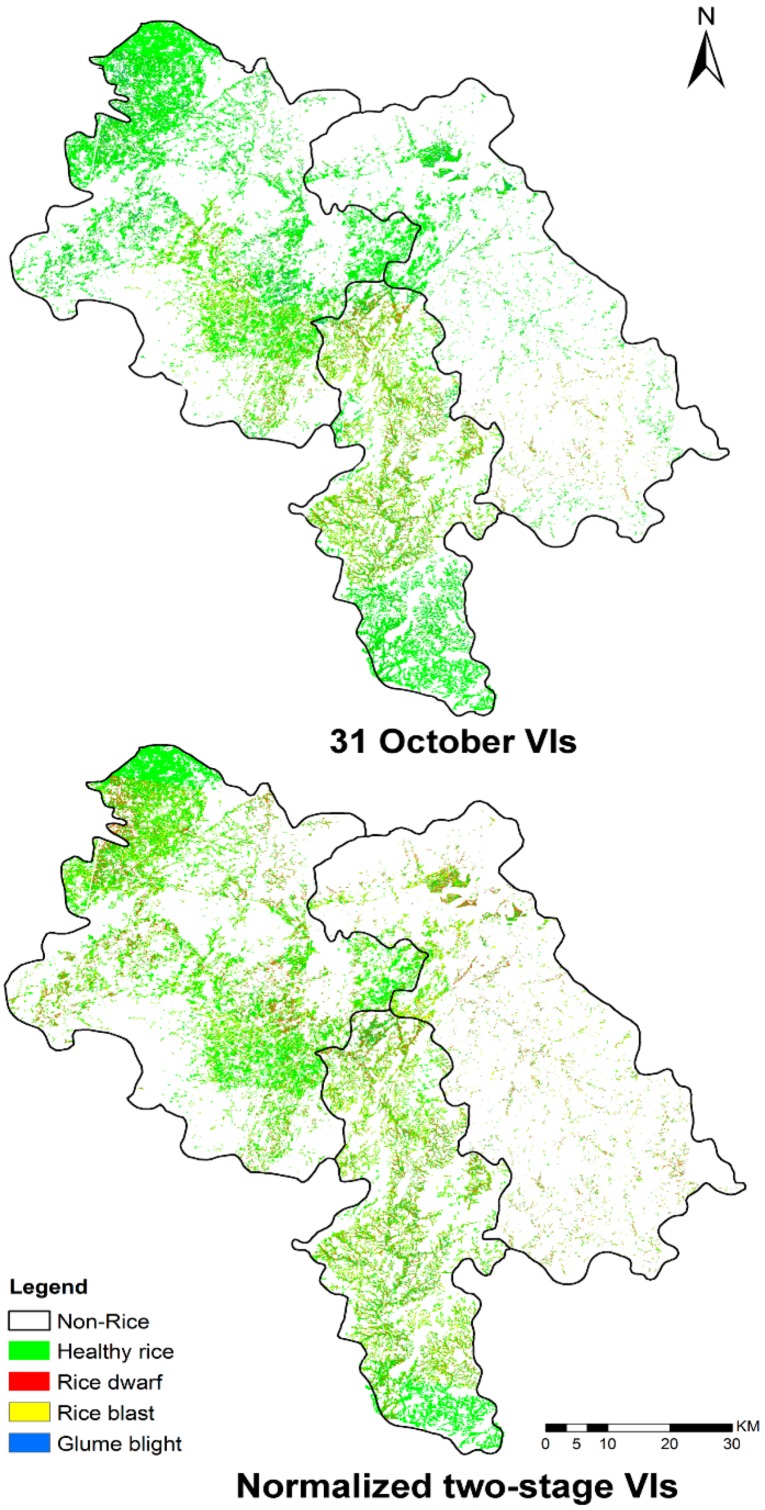
A map of healthy and diseased rice based on the PLS-DA classifier from normalized two-stage and single-date VIs.

The normalized two-stage VIs were used as input variables for building the feature space and producing disease distribution maps based on the proposed PLS-DA mapping frame (Figure 4). The spatial distribution of different diseases produced by the damage map was generally consistent with our field surveys. Thus, the damage caused by rice dwarf occurred in the center of Yulin City, north of Beiliu City, and north of Luchuan County. The damage caused by rice blast occurred in most of Yulin City, north of Beiliu City, and north-center of Luchuan County. The damage caused by glume blight was identified in the north of Beiliu City and Luchuan County. The disease infestations that occurred in the northern edge of Yulin City were confirmed through telephone interviews with the local plant protection department. The PLS-DA model was based on single-date VIs (calculated from the October 30 images) and a damage map was produced for comparison (Figure 4). The map aided in examining whether the normalized two-stage VI PLS-DA model had a significant improvement over the single-date image. The results revealed that the diseased area produced by single-date VIs was significantly less than that on the normalized two-stage map, especially for the identification of rice blast and glume blight.

A confusion matrix and kappa value were calculated to provide a quantifiable classification assessment, shown in Table 5. The normalized two-stage VI model returned an overall accuracy of 75.62% (kappa = 0.47), 13.95% higher than the single-date VI model. The classification accuracy for individual diseases ranged from 64.29 to 87.1%. Rice dwarf disease featured the highest classification accuracy of the three diseases. By comparison, in Table 5, the single-date VI model produced more commission errors in the detection and classification of different diseases (kappa = 0.27). These results suggest that the infestation was underestimated by single-date VIs and their combinations, which resulted in a significant reduction in disease mapping.

**Figure 5:**
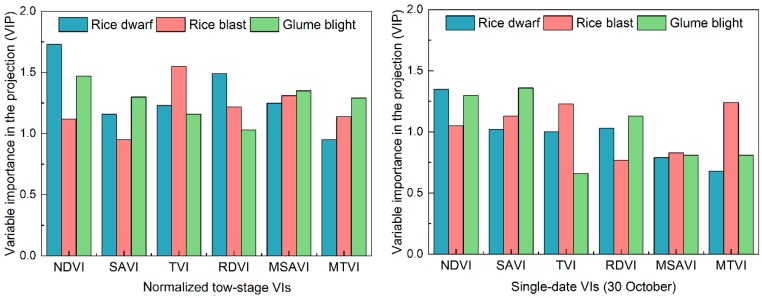
The importance of VIs for the detection of rice diseases as determined by the variable importance in the projection (VIP) method. Normalized two-stage and single-date VIs are projected as VIP scores.

Figure 5 illustrates the contributions of each individual spectral feature in the PLS-DA model by the VIP method. It suggests that the normalized two-stage VIs had a better performance than single-date VIs in detection the progressive development of internal and external symptoms during the disease detection. Thus, the sensitivity of normalized two-stage VIs to disease infestations enabled the capture of more pathological and biophysical evidence in damage mapping. For example, the normalized two-stage variation for NDVI, which represents the crop growth impacted by dwarf infestation, had the most significant contribution to the classification for the rice dwarf class (VIP = 1.73). The normalized two-stage SAVI, TVI, RDVI, and MSR had the contribution to represent corresponding biophysical variations caused by the infestations, including canopy morphology and chlorophyll variation on the pixel-scale. The normalized two-stage TVI performed best in the PLS-DA approach for the rice blast class with a VIP value of 1.47. The normalized two-stage NDVI, RDVI, MSR, and SIPI were also important variables in the PLS procedure (VIP > 1). The normalized two-stage NDVI, SAVI, TVI, MSR, and SIPI had similar contributions for the glume blight classification. In contrast, the contributions of single-date VIs were not significant owing to a similar spectral response pattern could be found, such as the NDVI for both rice dwarf and rice blast. 

## 4. Discussion

The damage produced by the rice dwarf virus primarily impacts plant growth, which thereby results in a significant reduction in leaf area and biomass [54]. Similarly, the glume blight infestation first affects the foliage and induces wilting and structural change in the canopy [55]. By comparison, the first symptom of rice blast infestation is dehydration and destruction of the pigmentary system, which leads to discoloration in appearance and a series of physiological and biochemical variations in leaves [56]. Changes in external architecture and internal biophysical parameters provide evidences for remote detection of diseases infestations. PlanetScope satellite data products collected high spatial resolution (3-m) imagery in broad blue, green, red, and NIR spectral bands. Among the four original bands, the NIR band showed evident difference between healthy and diseased rice, which was associated with variation in canopy variations. In this study, the selected VIs exhibit great performance on separating healthy rice from the rice infected with different diseases. These VIs enhance the original spectral response from different aspects. It was also noted that all of the VIs contained a NIR band, which thereby had the potential to be sensitive to the changes in canopy parameters at the regional scale, such as LAI and green biomass, and might explain their good performance in detecting diseases infestations. This finding is consistent with Qin et al. [57,58]’s studies.

Compared with the single-date VIs, the novel proposed normalized two-stage VIs performed better on characterizing the biophysical and canopy structure variations caused by disease infestations (Table 3 and Table 4). It is noteworthy that, apart from the disease infestations, there were other factors leading to responses of the same spectral features. Thus, within a single-date scene, the spectral features would not only respond to disease infestations, but also fluctuate following the phenological differences, cultivation differences, and plant condition diversities between fields [59]. The bi-temporal spectral variations characterized by the normalized two-stage VIs and the high spatial resolution (3 m) provided by the PL imagery helps to eliminate field anomalies other than the disease infestations and mixed pixel effects. For example, among the identified features, the normalized two-stage NDVI showed stronger sensitivity to rice dwarf for disease mapping, which was associated with variation in canopy morphology driven by change in leaf area. Normalized two-stage TVI performed better on rice blast classification owing to its sensitivity of radiant absorption of chlorophyll. And the normalized two-stage NDVI and MSR provide significant contributions for glume blight classification, which were highly correlated with canopy structure. These spectral responses extracted by the normalized two-stage VIs may explain the sound improvement in detecting different diseases. 

From the perspective of classification, this study revealed the potential of normalized two-stage VIs for accurately classifying rice diseases. PLS-DA provides an ideal framework for the combined purpose of integrating spectral features and pathological mechanisms. For instance, PLS-DA successfully reduced the redundant information and collinearity effect hidden in the input feature space. Furthermore, PLS-DA provides valuable information on important spectral features, based on the VIP approach. The normalized two-stage VI based PLS-DA model produced a better damage map and lower commission error for specific disease classification. In contrast, the classification produced by single-date VIs (30 October) had a reduced performance. The analysis of important features determined by the VIP procedure has shown that the highest scoring structures for the classification were consistent with the pathological progress of certain diseases. For example, the VIs that were sensitive to plant growth (such as the normalized two-stage NDVI, SAVI, MSR) made more contributions to the identification of rice dwarf. The normalized two-stage TVI, RDVI, and SIPI selected by VIP scores features a high correlation to pigments and chloroplast variations and indicated their relative importance in discriminating rice blast. The VIP method captured the typical symptoms of specific diseases and was important for monitoring and discriminating between diseased species. 

**Figure 6:**
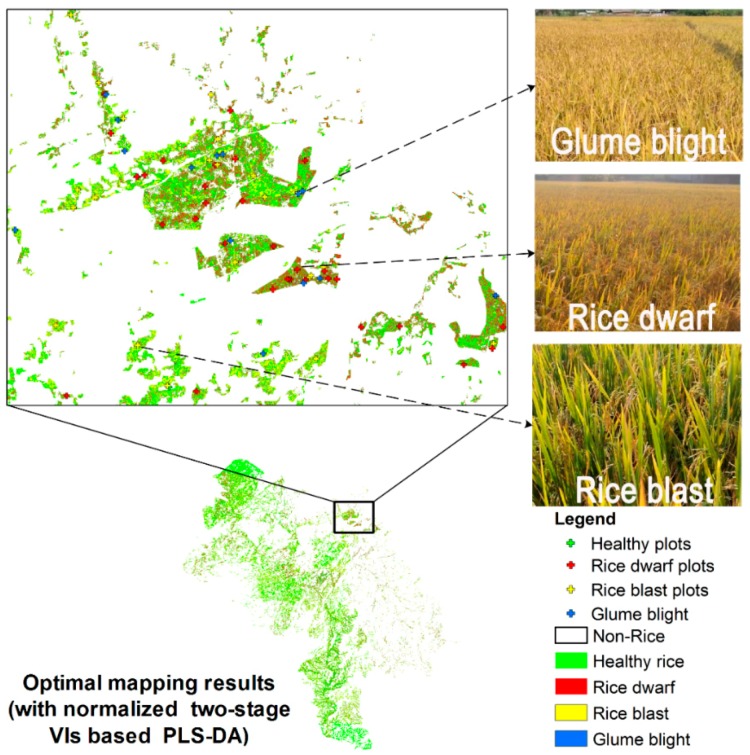
Mapping results of rice diseases in a sub-region based on the optimal normalized two-stage VIs based PLS-DA.

The results obtained from PL imagery revealed finer spatial detail at a 3 m spatial resolution and helped to resolve the heterogeneity in different disease infestations. For example, based on field surveys, the greatest concentrations of disease were highlighted and mapped using the optimal normalized two-stage VIs based PLS-DA model (Figure 6). The pixel-based classification of the diseases was highly consistent with our field observations which confirmed the effectiveness of the method (the photos from field surveys are provided in Figure 6). In contrast to manual interpretation, the proposed model aided in eliminating error and increasing the consistency and reliability of disease detection and discrimination for large areas. More importantly, our method provided a fast and effective way to assess the impact of diseases ranging from a single point observation to an entire region. This approach proved to be effective for assessing losses in crop yields. Our study successfully obtained damage information on rice diseases for the disease outbreak in the autumn of 2017 in Yulin City, Beiliu City, and Luchuan County. This information was forwarded to the Plant Protection Department in Guangxi and to insurance companies for agricultural management and damage assessment.

Overall, the novel developed normalized two-stage VIs based PLS-DA model performed better in monitoring and classification of diseases on rice based on the PlanetScope satellite image data, with an acceptable accuracy of 75.62%. On one hand, we expect PlanetScope’s high spatial resolution imagery to cover gaps in finer scale disease detection. On the other hand, its high revisiting cycle made it possible to mosaic and composite cloud-free imagery from the continues dates. However, because of mountain-dominated topographic conditions, rice fields are small and scattered, and it is still impossible to eliminate completely the influence of mixed pixel problem. Additionally, the error from noises from the co-registration between images will also bring uncertainty in the modeling processes. Hyperspectral data is capable of partly overcoming the limitation of mixed pixels, and providing more detailed spectral information for disease detection [60], but the high-dimensional information provided by the hyperspectral sensors always raises the computational complexity and cost. Therefore, our future research would investigate whether the hyperspectral satellite data could provide more information on diseases detection and eliminating the mixed pixel problem, and further improve the accuracies in disease species classification. Furthermore, by assimilating the high spatio-temporal resolution, multispectral data and the moderate resolution, hyperspectral data, we expect to develop time- and cost-effective strategies for early detection and monitoring of diseases before the specific symptoms become visible.

## 5. Conclusions

Monitoring of crop diseases at a regional scale is of practical importance for agricultural management and insurance claim. This study has contributed to the detection and mapping of different rice disease species at fine spatial scales. Firstly, a set of normalized two-stage VIs were developed to characterize the progressive development of disease infestations, subsequently, based on these satellite-derived spectral features, three kinds of rice diseases (i.e., rice dwarf, rice blast, and glume blight) were classified and mapped. Additionally, the study involved the first use of combined bi-temporal PL imagery. Our findings suggest that the normalized two-stage VIs can be used to identify the difference between the progressive developments of the various infestations on rice. Furthermore, this method can make optimal contributions in fitting the PLS-DA model to produce a reasonable disease map with an overall accuracy of 75.62%. The potential application of the normalized two-stage VI PLS-DA approach facilitates loss assessment for the agricultural insurance industry by examining spatial damage information caused by diseases. This includes the identification of suspected areas and deploying experts to direct prevention operations. Future studies should explore the use of VHSR imagery with short-wave infrared band (e.g., WorldView-3), which can produce more information for characterizing the pathological progress of disease infestations and for developing strategies for early detection and monitoring of diseases before the symptoms become visible. In this domain, more efforts and studies are needed to improve the performance and robustness of disease mapping techniques.

## Figures and Tables

**Table 1:** The parameters and information for the selected PlanetScope imagery.

Parameters	Information
Sensor	PlanetScope
Acquisition date	21 August 2017 and 30 October 2017
Orbit altitude	475 km
Spatial resolution (m)	3
Revisit time (days)	1
Wavelength range (nm)	
Band 1	Blue: 455–515
Band 2	Green: 500–590
Band 3	Red: 590–670
Band 4	NIR: 780–860
Signal-to-noise ratio (SNR)	68.8

**Table 2:** The vegetation indices used for classifications in this study, with red band, NIR band, and green band denoted as R_R_, R_NIR_, and R_G_, respectively, for the Planet Satellites.

Definition	Related Bands and Equations	Sensitive to	Reference
Normalized difference vegetation index, NDVI	(R_NIR_ − R_R_)/(R_NIR_ + R_R_)	Green biomass	[47]
Soil-adjusted vegetation index, SAVI	(1 + *L*) × (R_NIR_ − R_R_)/(R_NIR_ + R_R_ + *L*); *L* = 0.5	Canopy structure	[48]
Triangular vegetation index, TVI	0.5 × [120 × (R_NIR_ − R_G_) − 200 × (R_R_ − R_G_)]	Radiant absorption of chlorophyll	[49]
Re-normalize difference vegetation index, RDVI	(R_NIR_ − R_R_)/(R_NIR_ + R_R_)^0.5^	Vegetation coverage	[50]
Modified Simple Ratio, MSR	(R_NIR_/R_R_)/(R_NIR_/R_R_)^0.5^	Leaf area, Biomass	[51]
Structural Independent Pigment Index, SIPI	(R_NIR_ − R_B_)/(R_NIR_ − R_R_)	Pigments content	[52]

**Table 3:** A comparison of the independent classification abilities of selected spectral features.

	Means of Normalized Two-Stage VIs				Means on 30 October			
	Healthy Rice	Rice Dwarf	Rice Blast	Glume Blight	Healthy Rice	Rice Dwarf	Rice Blast	Glume Blight
NDVI	**	**	**	**	**	*	**	*
SAVI	***	*	***	*	*	**	**	*
TVI	**	*	***	**	**	*	**	*
RDVI	**	**	***	**	*	*	**	*
MSR	**	**	***	**	*	*	**	*
SIPI	***	***	**	***	*	*	**	**

Note: *** classification accuracy ≤ 60%, ** 30% ≤ classification accuracy < 60%, * classification accuracy < 30%.

**Table 4:** The sensitivity of normalized two-stage and single date VIs based on ANOVA analysis.

	Means of Normalized Two-Stage VIs				Means on 30 October			
	Healthy Rice	Rice Dwarf	Rice Blast	Glume Blight	Healthy Rice	Rice Dwarf	Rice Blast	Glume Blight
NDVI	0.032 **	0.014 **	0.058	0.065	0.078 *	0.018 **	0.08 *	0.102
SAVI	0.047 *	0.046 *	0.036 *	0.079	0.032 *	0.031 *	0.166	0.052 *
TVI	0.032 *	0.02 **	0.04 *	0.012 **	0.037 *	0.104	0.142	0.051 *
RDVI	0.01 *	0.009 **	0.045 *	0.066	0.048 *	0.074 *	0.125	0.091
MSR	0.057	0.095	0.027 **	0.091	0.073	0.125	0.077	0.054 *
SIPI	0.039 *	0.027 *	0.087	0.036 *	0.056 *	0.124	0.145	0.09

Note: * indicates the different significance at 0.95 confidence level. ** indicates the different significance at 0.99 confidence level.

**Table 5:** The confusion matrices and classification accuracies produced by normalized two-stage VIs and single-date VIs with the PLS-DA method.

Predicted Class	Healthy Rice	Rice Dwarf	Rice Blast	Glume Blight	User’s Accuracy (%)	Overall Accuracy (%)	Kappa Coefficient
Normalized two-stage VIs							
Healthy rice	54	0	6	2	87.1	75.62	0.47
Rice dwarf	4	60	5	9	76.92		
Rice blast	11	4	48	5	70.59		
Glume blight	5	8	2	27	64.29		
Producer’s accuracy (%)	72.97	83.33	78.69	62.79			
single-date VIs							
Healthy rice	47	3	8	4	75.81	61.67	0.27
Rice dwarf	8	48	5	17	61.54		
Rice blast	16	6	39	7	57.35		
Glume blight	7	11	4	20	47.62		
Producer’s accuracy (%)	60.26	70.59	69.64	41.67			
